# A Systematic Review on Strategy Training: A Novel Standardized Occupational Therapy Program for Apraxia Patients to Perform Activities of Daily Living

**DOI:** 10.7759/cureus.23547

**Published:** 2022-03-27

**Authors:** Arsalan Moinuddin, Khursheed Faridi, Yashendra Sethi, Ashish Goel

**Affiliations:** 1 School of Sport and Exercise, University of Gloucestershire, Gloucester, GBR; 2 Department of Anatomy, National Institute of Medical Sciences (NIMS) & Research/NIMS University, Jaipur, IND; 3 Department of Medicine, Government Doon Medical College, Dehradun, IND; 4 Department of Physiology, Government Doon Medical College, Dehradun, IND

**Keywords:** occupational therapy program, motor learning models, activities of daily living (adl), apraxia, strategy training

## Abstract

Apraxia is a cognitive-motor planning disorder that expresses itself as an inability to perform purposeful and skilled movements in the absence of sensory or motor loss and hampers patients' ability to perform activities of daily living (ADL). ADL is a set of everyday tasks required for personal care and independent living, executed through a complex interaction between sensorimotor integration and motor learning. We have designed a ‘Strategy training’ program for apraxia patients by reviewing the existing clinical trial literature on the above-said topic per the Preferred Reporting Items for Systematic Reviews and Meta-Analyses (PRISMA) guidelines. Strategy training is an evidence-based standardized occupational therapy program to improve ADL in apractic patients by teaching them a variety of compensatory strategies to combat impairment and improve activity performance. Three basic steps of strategy training include: 1) initiation-development of an action plan, 2) execution-performance of the plan, and 3) control-assessment and result of action performed. The patient group suggested for strategy training comprises post-stroke (past 20 weeks) apraxia patients aged 40-90 years of both genders, highly motivated and fit to perform ADL. After preliminary assessment, ‘strategy training’ will be specifically executed through an exclusively visual feedback approach in which apraxia patients learn eight ADLs in 8 weeks (three sessions of 30 minutes/week for 8 weeks). They practice two ADLs for 15 minutes each in every session, thus a total of six sessions will be allocated to learn two ADLs simultaneously followed by the next set of ADLs. Strategy training for brushing teeth is described in detail to show how each step of this training program is implemented for a specific ADL. As this strategy training program is based on individual care, attention, and augmenting motivational aspects, it is expected to teach patients compensatory strategies to learn and perform ADL more smoothly, swiftly, and most importantly independently. The program is not aimed at treating clinical motor symptoms of apraxia per se but to help patients function more independently post apractic motor impairment.

## Introduction and background

Introduction

Apraxia’ Disease

Apraxia’ stems from Greek which means ‘without action’ and clinically manifests as decreased ability to do purposeful skilled movements [1,2] . ‘Limb apraxia’ is a cognitive-motor planning disorder that expresses as an inability to perform purposeful and skilled movements in the absence of sensory or motor loss, or disorders of memory and comprehension [3]. It hampers the patients’ ability to perform activities of daily living (ADL) and/or show communicative gestures and is present as one of the consequences of diseases such as stroke, Alzheimer’s, corticobasal degeneration, and dementia [2,3]. Apraxia is not caused by any physical disability; it is rather a result of higher-order cognitive neurological impairment (especially parietal lobe) at the level of three systems: sensory or perceptual system that processes visual and auditory information, conceptual system which gathers knowledge, and the production system which organizes, plans, and executes motor action [4]. Apraxia typically manifests as an inability to both pantomime (show gesture from memory to verbal cue) and mimic a visually demonstrated gesture. The two subtypes of apraxia are: ideational apraxia: diminished performance of skilled activity because of lack of conceptualization of action and ideomotor apraxia: impaired initiation and execution of planned movement sequences. Ideational apraxia patients may smear toothpaste on face or eat soap whereas ideomotor apraxia patients exhibit clumsy movements such as picking up a bottle with a pinch grip (instead of cylindrical grip). However, irrespective of the variant, the patient fails to implement his plan of performing a motor movement optimally [5]. To avoid any sort of confusion caused by the differential use of terminologies for apraxia, we will stick to the term ‘apraxia’ which encompasses both ideomotor apraxia and ideational apraxia throughout our discussion. The paucity of studies on apraxia is due to the assumption that it does not hamper daily life significantly, however, apraxia exhibits negative impact on both ADL and overall rehabilitation recovery. A review of literature on management of apraxia reveals ‘10’ treatment approaches adopted by clinicians, therapists, and researchers to treat it. They include multiple cues, error reduction, six-stage task hierarchy, conductive education, strategy training, transitive/intransitive rehabilitative treatment, errorless completion + exploration training, errorless completion, and exploration training [2].

Activities of Daily Living (ADL)

ADL is a set of everyday tasks required for personal care and independent living. It is classified as 1) domestic ADLs, 2) extra-domestic ADLs, and 3) physical self-maintenance (PSM), most commonly, tasks such as bathing, dressing, toileting, grooming, and eating [1] come under PSM ADL, and for the sake of clarity, we will only refer to this category throughout our discussion. ‘ADL’ can be defined as per the different motor learning models:

ADL on five one-dimensional classification taxonomies: 1) Muscular involvement continuum: ADL involves both gross skills (large muscles: shoulder, arm, forearm) and fine skills (small muscles of wrist, hands, and fingers). It depends on the specific type of ADL. 2) Environmental influences continuum: ADL are closed motor skills that are performed in predictable environment with full control of both initiation of action and timing of movements. 3) Continuity continuum: ADL are serial discrete skills linked together in a movement sequence and perform in a specific order. 4) Pacing continuum: ADL are self (internally paced) as the learner can control the speed, timing, and rate of skill. 5) Complexity continuum: ADL constitute a simple skill as a small amount of information is processed with fewer decisions to take while performing the skill [6].

ADL on gentile’s 2D taxonomy: This approach implies that our every action is a result of complex interaction between the performer, task, and the environment. Specific type of ADL decides which grid of Gentile’s 2D taxonomy is suitable. Overall, for any ADL, from an ‘environmental viewpoint’, stationary regulatory conditions persist with internal variability absent/present allocating either row 1 or 2 to it. From an ‘action function’ perspective, body remains stable or moves with 1) ‘no’ object manipulation (column A) or 2) object manipulations (column B). Thus, depending upon the ADL type, scores are allocated from (1A-2D); e.g., brushing teeth (1B), from lying on bed to chair (1C), and so forth [6].

Fitts and Posner’s three-stage model, Dynamical System Model: In ‘strategy training’, two new ADL are practiced every session for a total of six consecutive sessions every 2 weeks (3 sessions/week). This means that a patient typically moves from ‘cognitive’ stage to ‘associative’ stage (Fitts and Posner’s 3-stage model) or from ‘novice’ to ‘advance’ (Dynamical System Model) by the end of the second week. However, strategy training does not provide specific guidelines for change in instructional technique with patient learning keeping these models’ transition perspective in mind [7].

Strategy Training

‘Strategy training’ is an evidence-based standardized occupational therapy program originally designed by Van Heugten to supplement the existing rehabilitation program for apraxia in stroke patients [5]. The main aim of the program was to improve ADL in apractic patients by teaching them a variety of strategies that help them perform ADL more efficiently and independently within the constrain of the leftover function and not striving for functional recovery. The primary focus of strategy training was to coach patients’ compensatory approaches to combat impairment and improve activity performance despite apraxia either externally or internally. External compensation can be given in the form of auditory or visual cues to carry out an ADL, if he or she has plan sequencing problems related to that particular task. This model encompasses 1) an internal compensation strategy that is focused on conscious self-verbalization and 2) an external compensation strategy, i.e., showing pictures of how to accomplish a task. These two cornerstones of strategy training approach are employed when apraxia patient fails to sequentially follow steps for task completion [2]. This model is adapted by both Van Heugten and Donkervoort in their strategy training regimes of three and five ADLs, respectively [5]. ‘Strategy training’ is based on ‘Occupational Therapy Practice Framework’ model of motor behavior which depicts complex interface between the personal (sensorimotor, cognitive, psychosocial) and environmental (physical, cultural, socioeconomic) characteristics. The phases of the training are centered around the fact that CNS interacts concomitantly with both personal and environmental systems when a person pursues a functional goal.

Objectives

The current systematic review is aimed to focus on ‘strategy training’ and construct a practice session design for apraxia patients to perform PSM ADL to lead a hassle-free living post-stroke.

## Review

Methods

Data Sources, Searches, and Article Selection

**Figure 1:**
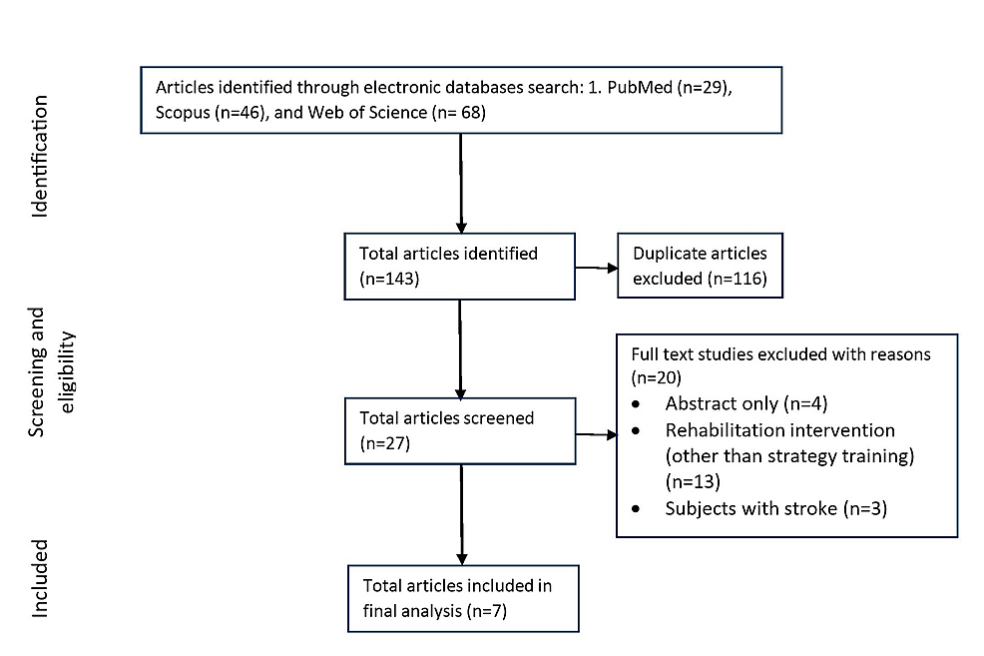
Flowchart depicting the inclusion and exclusion criteria per the PRISMA guidelines PRISMA: Preferred Reporting Items for Systematic Reviews and Meta-Analysis

The reporting of this review was guided by the standards of the Preferred Reporting Items for Systematic Reviews and Meta-Analysis (PRISMA). The literature search for this review has been limited to electronic databases (PubMed, Web of Science, and SCOPUS) and was searched employing the keywords: (strategy training OR occupational therapy) AND (apraxia) AND (activities of daily living). Additionally, we’ve looked into the reference list of trials and reviews identified in the final analysis. Our search was limited to English language studies published between inception and Sept 2021. A detailed step-wise description of the searched methodology is depicted in Figure 1.

Patient Characteristics

The patient group suggested for ‘strategy training’ comprises apraxia patients aged 40-90 years of both genders (males and females). Per the anthropometric measurements, strategy training will not specifically take the weight of the patient into consideration while training, however, overweight and obese individuals have generalized difficulty in learning a few ADL tasks (e.g., taking bath). Typically, less flexible than healthy individuals as immobility/less mobility due to stroke reduces flexibility. A clinical diagnosis of stroke (ischemic stroke or hemorrhagic stroke in the past 20 weeks) and apraxia (inability to carry on ADL either fully or partially and NOT due to sensory or motor deficit, comprehension deficit, and attention or memory deficit. Other neurological impairments if present will not impede ADL in one way or other. Patients with visual or hearing deficit, psychiatric illness, drug or alcohol addiction, and any sort of learning disorders are excluded from this study. Patients should be fit enough to perform ADL which means that any weakness or coordination impairment if present will not hamper their ability to perform ADL from a motor system perspective. They should be highly motivated to learn ADL as close to the normal as possible with preferably no element of fear present. Their skill level to do motor activities cannot be determined like normal healthy individuals as their praxis system is damaged due to disease. Therefore, a two-stage ‘preliminary assessment’ system will be used to assess their skill level before the start of strategy training (discussed later). ‘Strategy training’ is designed with a thought that apraxia patients learn ADL as naïve sensorimotor learners. The therapist making them perform ADL should be fully acquainted with the patient’s language.

Specifics of ‘Strategy Training’ Task

Only the patient and therapist will be present at the time of training because ADL are typically performed alone even by normal healthy individuals. This will also avoid undue anxiety which may creep in in the presence of others that hampers overall learning. Strategy training will help participants learn ADL slowly over time. The training will include visual, auditory, and tactile stimuli to learn ADL, however, the reliance will be more on visual stimuli just like a normal individual. The training will be given utilizing the same objects a person will use after training to perform ADL in the future. ADL may be modified or simplified impromptu depending upon the patient’s response to learning, e.g., while brushing teeth, toothbrush and toothpaste will be placed in the order of use, with lid of toothpaste open in order to reduce coordination requirements [5].

Environmental Factors in ‘Strategy Training’

Strategy training will be carried out in a closed predictable environment where a learner has full control over his actions and timing of movements. Each ADL will be performed in a different environment depending upon the ADL itself, e.g., brushing teeth in a bathroom, combing hair in a dressing room, and so forth. Only the patient and therapist will be present at the time of training because ADL are typically performed alone even by normal healthy individuals. Strategy training for all the ADL is practiced in either a bedroom, living room, or bathroom in a thermoneutral environment (75°F) in a bright day or artificial light. The idea is to train the participant in the same environment where he will use his skill in future. The instructor will make sure that the participant will be encouraged (verbal cheering, clapping) and no disruptions take place (e.g., audio, visual, someone trespassing into the practice room) throughout the training session.

Practice Design

Preliminary assessment:It will be done through choice of activity, assessment of the level of disabilities in ADL, and utilizing Barthel Index. 1) Choice of activity [8]: Activities will be chosen based on their priority level to the patients. This relevance in choosing ADL to be learned is decided based on a ‘checklist’ filled by the patient and a personal ‘verbal discussion’ with the patient. The purpose of the ‘check list’ is to sort out the roles played by major ADL in patients’ lives. The roles will be checked twice-first, depending upon the ADL execution in the past, present, and future, and, second, depending upon how valuable or important is the ADL to the patient (Table 1) [9]. 2) Assessment of level of disabilities in ADL [9]: The trainer will allocate grade to the patient on a point scale from 1 to 10 for the ADL observed with 1 given if the task was extremely hard/impossible to perform and 10 if the task was performed normally and independently (Table 2) [10]. 3) Barthel Index [10]: It is a measure of functional independence for ADL that will be used before and after the strategy training both to assess the level of impairment as well as improvement after training. It helps in identifying the most relevant ADL impairment and its severity. It is scaled 0-100 and includes activities such as feeding, bathing, grooming, dressing, bowel control, bladder control, toilet use, transfers (bed to chair), mobility on level surfaces, stairs, etc. [1,2].

**Table 1:** Preliminary checklist for the choice of ADL ADL: activities of daily living

Name			Age	Date
Sex: M/F				Retired: Y/N
Marital Status				
Part—I Execution in the past, present, and future				
Activity	Past	Present	Future	Therapists Notes
Part—II Value or importance of role				
Activity	Not at all valuable	Somewhat valuable	Very valuable	Therapists Notes

**Table 2:** Grade assessment of level of disabilities by the trainer

Date				
S. No	Self-Care Task	Task specifics (for scoring purpose)	Score (1-10)	Comments
1.	Drinking	Not spilling 1/8^th^ of hot liquid-filled cup		
2.	Comb hair	Looks neat on completion		
3.	Wash face/hands	open/close tap, hands *maneuvering* water on face		
4.	Make up or shave	Looks neat on completion		
5.	Brush teeth	Remove toothpaste lid, put paste, open/close tap		
6.	Eating	Cheese on toast		
7.	Undress	Clothes, socks, and shoes taken off		
8.	Bed to chair	From lying on bed to chair (with arms)		
9.	Lavatory	Movement to bathroom, managing trousers, cleaning		
10.	Indoor mobility	Movement in different areas of house		
11.	Dressing	Putting on clothes, socks, and shoes		
12.	Wash in bath	Showing movements		
13.	Overall wash	Wash arm at basin		
14.	Floor to chair	From lying on bed to chair (without arms)		
15.	Outdoor mobility	Covering 50-meter distance		
16	In/out of bath	Dry bath		

**Figure 2:**
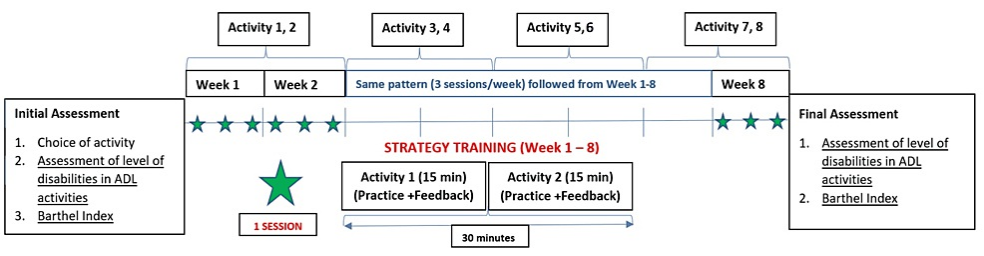
Overview of ‘Strategy Training’ study design ADL: activities of daily living

Overview of practice design (Figure 2): Two ADL will be selected every two weeks and each ADL will be practiced for 15 minutes during the 30 minutes session every day. With a total of three sessions scheduled for a week, a total of six sessions will be employed for practicing two ADL. This will be followed by choosing another set of two new ADL for the next 2 weeks and so on. Thus, a) total activities: eight ADL will be chosen (out of 16 listed), b) total duration of ‘strategy training’: 8 weeks, c) no. of sessions per week: 3, d) total no. of sessions in the ‘strategy training’ program: 8 × 3 = 24 sessions, and e) duration of each session: 30 minutes.

Detailed steps of practice design: A detailed elaboration of the steps of strategy training with ‘brushing teeth’ as an example quoted for each step is shown in Table 3.

**Table 3:** Elaborate description of ‘strategy training’ with ‘brushing teeth’ as an example quoted for each step

S. No			Steps of Strategy Training	Example of Brushing Teeth
	Initiation of activity	Instructions: When client has difficulty in initiating the task	Verbal command	“Get the toothbrush & toothpaste to brush your teeth”
			Transfer to environment optimum for task execution	“Get close to the washbasin to brush your teeth”
			Pantomime, pointing	Point toward toothbrush and toothpaste
			Mimic/demonstrate part of task himself	Mimic brushing teeth without and then with brush in hands
			Show pictures, video of initiation of the activity	Show picture/video of someone brushing teeth
			Jot down instructions	Write instructions of how to brush teeth with toothbrush and toothpaste
			Put objects near patient, then point, and then place in right sequence	Place toothpaste and toothbrush near the patient
			Initiate activity and then leave it for the patient to do	Position limbs to initiate brushing teeth
	Execution: Adequate performance plan	Assistance: When client has difficulty in executing the task	No verbal assistance needed	
			Verbal assistance, Recite steps of task, Name steps/objects, Task specifications	“Open the toothpaste lid, squeeze to get toothpaste out, put in on the brush, place brush in mouth and then move it in circular motion between and around teeth”
			Use gestures and mimics activity to assist patient	Continue assisting patient doing mimicry of brushing teeth throughout or as long as required
			Show pictures, video of most/all steps of the activity	Continue showing patient picture of someone brushing teeth as he continues to do it himself
			Physical Assistance (throughout the task): positioning of limbs, employing aids, start performing for the patient until he takes over	Get his limbs (arms, forearms, and hands) positioned to make him do whatever was recited in the verbal assistance above
			Takes complete charge of task	
	Control: Assessment and result	Feedback: When client has difficulty in controlling or correcting the activity to get the desired end result	No feedback necessary	
			Verbal feedback of result (KR: Knowledge of result)	Let the patient know that he has successfully completed the task
			Verbal feedback of performance (KP: Knowledge of performance)	
			Physical feedback to achieve the result	
			Video feedback recording watched and explained simultaneously to the patient	

Discussion

The discussion will elaborate motor learning factors that surround and influence ‘strategy training’ for apraxia.

Augmented Feedback

A combination of audio and visual feedback will be given to the participant after every ADL in the allocated 15 minutes time during each session. After the task, the recorded ADL will be shown to the patient and a verbal feedback on result (KR), performance (KP), and suggestions to improve will be given while watching the video with the patient. However, strategy training does not provide specific guidelines for change in this AF frequency with patient learning.

Modelling

Observational learning implies learning by watching and imitating another individual (model) and this is strongly implied during both ‘initiation of activity’ (therapist ‘mimic/demonstrate part of task himself’) and ‘execution of activity’ (therapist mimics activity to assist the patient).

Verbal Cues

Akin to modeling, verbal cues strongly influence strategy training for ADL both during ‘initiation of activity’ (therapist provides verbal command to initiate the task) and ‘execution of activity’ (therapist verbal assistance by reciting steps of task, and naming steps/objects). Attention cueing will be tried sometimes to make sure that the participant is paying maximal attention to a specific aspect of the skill while performing it as a whole. For example, while brushing teeth the learner will be focusing mostly on transferring paste onto the brush. The instructor will make sure that the participant will be encouraged (verbal cheering, clapping) while performing the task.

Distribution of Practice and Contextual Interference

Although ‘strategy training’ does not follow ‘blocked practice’ entirely (2 ADL at a time are used instead of 1), however, it is heavily inclined toward this approach as two ADL will be selected every two weeks and each ADL will be practiced for 15 minutes during the 30 minutes session every day. With a total of three sessions scheduled for a week, a total of six sessions will be employed for practicing two ADL. This will be followed by choosing another set of two new ADL for the next 2 weeks and so on. In contrast to ‘random practice’ this approach works better in acquisition (short-term) than retention (long-term). ‘Strategy training’ is low in contextual interference as different ADL are not mixed well while practicing and there is adherence to a set of two ADL at a time.

Part vs Whole Practice

Typically, as a task, ADLs are high in organization which means that subcomponents of the task/ADL are interdependent, i.e., performance of each part heavily depends upon the preceding component. They are low in complexity with not many subcomponents making up the skill. As a result, strategy training for ADL will focus on the whole practice instead of part practice.

Variability and Specificity of Practice

Typically, ‘strategy training’ works on ‘constant practice’ schedule and will not vary the conditions of practice much during practice as all ADL will be practiced in the same environment (bedroom, living room, or bathroom) where the learned skill will be used in future, and this approach alike ‘distribution of practice’ works well in acquisition (short-term) than retention and transfer (long-term). Although, some variability is seen after completion of eight ADL with one ADL learning somewhat influence the learning of another ADL (especially the ones with rather similar skills, e.g., ‘bed to chair’ and ‘floor to chair’). ‘Strategy training’ follows the ‘specificity of practice’ principle strongly and all ADL will be practiced in the same testing conditions (bedroom, living room, or bathroom) where the learned skill will be used in the future.

Limitations

The major limitation of this review are 1) the availability of only a few articles (2017-2020) that specifically address strategy training in apraxia patients, 2) all the research on ‘strategy training’ in apraxia patients is confined to western Europe constraining the generalizability of its findings, and 3) alluding ‘strategy training’ is limited in scope in terms of its conceptualization to post-stroke patients’ capacity to perform PCM ADL.

## Conclusions

Since this ‘strategy training’ program is based on individual care, attention, and scientific augmentation of general motivational aspects, it is expected to teach patients compensatory strategies to learn and perform ADL more smoothly, swiftly, and most importantly ‘independently’. This program is not aimed at treating clinical motor symptoms of apraxia per se but to help patients function more independently post apractic motor impairment. However, occupational therapists may observe a sort of ‘ceiling effect’ during ‘strategy training’-patients who perform ADL rather independently before the program may not exhibit much progress. As such, there remains a need to determine how long ‘strategy training’ will sustain post-therapy in apraxia patients, keeping in mind their gradual-worsening of motor impairment with advancing age. Based on the limited existing literature, despite the everlasting presence of apraxia this therapy program is anticipated to wane off motor impairments associated with ADL and help enable these patients to function in a more autonomous fashion.
